# Which Contributes to Clinical Performance: Academic Output or Person–Environment Fit?

**DOI:** 10.3389/fpubh.2022.801917

**Published:** 2022-03-04

**Authors:** Minye Dong, Yuyin Xiao, Chenshu Shi, Wu Zeng, Fan Wu, Guohong Li

**Affiliations:** ^1^School of Public Health, Shanghai Jiao Tong University School of Medicine, Shanghai, China; ^2^Center for Health Technology Assessment, China Hospital Development Institute, Shanghai Jiao Tong University, Shanghai, China; ^3^Shanghai Winking Entertainment Corporation, Shanghai, China; ^4^Department of International Health, Georgetown University, Washington, DC, United States; ^5^Shanghai Medical College, Fudan University, Shanghai, China

**Keywords:** qualification certification, career progression, performance assessment, clinical performance, academic output, person-environment fit, promotion

## Abstract

**Background:**

The measures put in place by health authorities to ensure the professionalism of doctors are important. Hospitals in China have included academic outputs in the promotion criteria to incentive medical clinicians to engage in scientific research so that to improve job performance (JP). However, such practice disproportionally focuses on academic outputs but ignores the force of needs fulfilled brought by intrinsic incentive. This study aims to discuss the realistic problem regarding the promotion mechanism and the potential drivers to clinical JP.

**Methods:**

This study was based on multi-source data collection on clinical performance from electric medical record (EMR), person-environment (P–E) fit from the survey, and academic output from personnel files of ward clinicians (*n* = 244) of general public hospitals who sought for career progression in Shanghai in 2020. Independent-Sample *t*-test and chi-square test were used for comparison of two sample means or constituent ratio between promoted and not promoted clinicians. Linear multilevel regression was conducted to examine the relationship between clinical performance and academic outputs and P–E fit.

**Results:**

Clinicians who were promoted were more productive in producing academic outputs than those who were not (*t* = −5.075, *p* < 0.001). However, there was no difference in clinical performance between the two groups (*t* = −1.728 to 0.167, *p* > 0.05). The regression showed that academic outputs were not related to clinical performance, while higher P–E fit was associated with the improvement of various clinical performances.

**Conclusion:**

This study shows that P–E fit plays a more important role in facilitating clinical performance than academic performance and highlights the importance of intrinsic motivation of clinicians in achieving clinical performance.

## Introduction

The controls put in place by health authorities to ensure the professionalism among licensed doctors are paramount in influencing the ultimate quality of care they provide (1). In China, licensed doctors have to pass a performance assessment in placed by the health department. The career ladders of Chinese clinicians have four levels, including residents, attending doctors, associate chief doctors, and chief doctors. The former two levels are examination based, and doctors are given the title primarily based on the test of their skills and application of clinical knowledge. The latter two levels are appraisal-based and involved in the comprehensive assessment of overall achievement including clinical, scientific, and innovative competency. Health authorities would annually accept applications from clinicians at different levels and promote the standouts through qualification appraisal. Promotion always serves for the input targets in performance information used to keep perceived autonomy, competence, and relatedness of the professionals in public sectors (2).

One prominent promotion standard for clinicians in China is the requirement of academic publications for promotion. It is assumed that the engagement of clinicians and healthcare organizations in research would enhance healthcare performance (3, 4). In the past decades, China has encouraged clinicians to partake in research. Therefore, the government has issued various preferential policies such as financial incentives, career advancements, and resources tilt to support the development of scientific research. Hospitals and hospital regulatory agencies have also incorporated indicators such as the number of journal articles published and research projects (RP) awarded into career progression standards for clinicians, so as to encourage them to conduct scientific research. Such output control of the management control system incentivizes individuals to engage in a particular behavior (e.g., journal publications) and rewards individuals (e.g., promotion) for producing specific results (5). Under such circumstances, promotion often serves as extrinsic incentives and rewards of research excellence (6).

For individuals, promotion not only signifies career identity and success, but also grants permission to perform more challenging tasks and make autonomous decisions. To get promoted, clinicians in China under the current promotion standard are motivated to perform both clinical activities and academic research. As a result, it has brought out a boom in producing academic outputs (e.g., journal publications) in the past decades in China. The number of research papers in China has grown rapidly, far exceeding the world average level. It is reported that from 2006 to 2010, the average annual growth rate of SCI papers in China was 19%, compared with only 4% globally (7). However, such practice disproportionally focuses on academic outputs, an intermediate objective of achieving high clinical performance, does not necessarily improve health outcomes. There is in fact an inflation for getting research outcomes, regardless of the real contribution of these publications.

Besides, routine clinical tasks of doctors may be affected by multitasks demands when scientific research becomes compulsory for every doctor. Although multitasking can improve efficiency, studies also show that multitasking may produce worse performance when polychronicity levels of individuals are not congruent with the environment or job (8, 9). Vroom has pointed out that there should be a fit between characteristics and attitudes and the workplace of individuals (10). “Fit” plays a key, but often ignored, role in the relationship of motivation and performance (11). However, there are few studies on person–environment (P–E) fit among clinicians on their job performance (JP).

### Assessment of Clinical JP

Job performance is a complex multidimensional construct, with many different meanings depending on who evaluates it, how it is evaluated and what aspect is being evaluated, etc. A meta-analysis indicates that objective and subjective performance measures should not be used interchangeably (12). Self-evaluations from employees often suffer from inflated bias and they seldom agree with the ratings of superiors (13). Besides, evidence shows that supervisor evaluations are more of a reflection of the quality of the interpersonal relationship between the superior and subordinate rather than the quality or quantity of the performance of the subordinate (14) and objective evaluations can mitigate potential self-reporting biases.

In healthcare specifically, despite no unified model yet, several constructs of clinical JP have been developed. For example, the Clinical Value Compass (CVC) (15) is a 4-point formal framework to measure the objective JP of clinicians. It measures functional, costs, clinical outcomes (COs), and patient satisfaction and has been used to examine the impact of assessment and feedback on the clinical performance of physicians (16) and to measure the quality of healthcare (17, 18). Another wide-used PMS instrument developed by Cavalluzzo and Ittner (19) captures similar four results-oriented performance measures at the hospital level: quantity and quality of products or services provided, operating efficiency, and customer satisfaction.

In addition, measuring instruments such as CVC also suggest the operational definition of measures based on information from the medical, administrative financial, and patient records. Functional dimensions focus on the health or risk status. It can also be interpreted as the volume and technical difficulty of services the clinician provided. Cost dimension focuses both on time and cost [e.g., hospital charges, length of stays (LOSs), and readmissions]. CO and satisfaction are the measurement of service quality, outcome indicators including mortality, morbidity and complication, etc. Overall, the value of health services can be represented as a function of quality, costs, and volume overall (20), see in Equation (1). Such function determines the direction of indicators, that quality and volume are positively related to performance and cost is negatively related to performance.


(1) $$\begin{array}{r} Value\;of\;health\;services\;=\;\frac{Quality}{Costs}\times Volume \end{array}$$

### Nature of Academic Outputs and Clinical Performance

Scientific research plays a significant role in advancing clinician development. The importance of research to healthcare was demonstrated in the charter of Britain's National Health Service (21), which advocated for increasing research capacity to improve the quality of care in hospitals (22). A JAMA study claimed that creating an educational environment supporting the research of residents helped to develop analytical skills and promote critical thinking, which further promoted evidence-based medicine and quality patient care (23). Therefore, the focus of the discussion on research in health care settings is not whether it is necessary for clinicians to engage in scientific research, but to what extent academic outcomes can contribute to improve the ability of clinicians and further improve the effectiveness of clinical treatment.

The evidence to support a positive association between the engagement in research and clinical performance was less strong than previously thought. Although some empirical studies found that conducting research studies were associated with the level of hospital improved clinical performance as reflected in increased healthcare quality (Care Quality Commission scores) (24), reduced deaths (22, 25), 5-year survival (26), and reduced LOS (27). Some other studies had suggested that the effectiveness often depended on the context in which they operated (4), different strategies may yield different consequences (4, 28). For example, a systematic review distinguished the influence of research on different kinds of clinical performance, namely clinical process and outcome. The study found that participation in clinical trials only contributed to better adherence to guidelines in the clinical process, but the effect on CO of patients was uncertain (29). A study indicated that the positive relationship between scientific research and clinical practice was predominantly driven by intervention studies rather than observational studies activities (24). Another study discussed the differential effect of high-quality vs. high-volume academic research on medical practice. The results suggested that high-quality research produce better results in terms of practical outcome than high-volume but low-quality research (30). At the individual level, most of the other studies at individual level focus on how clinicians balance different workloads between clinical, educational, research, and administrative duties and the burnout caused by imbalance (31). For example, studies found the increases in the clinical workload might diminish research outputs (32).

### The Nature of P–E Fit and JP

The “fit” or “congruence” between what individuals want and what they get from their work and organization has been widely studied (33). The P–E fit theory has become increasingly popular in explaining how individuals think and behave within an organization (34). If the characteristics of an individual are well-matched with the institutional environment, the individual tends to have a higher level of internalization in translating organizational goals into his/her personal priorities and concentrates on what is important for the organization (35). It would lead to positive personal outcomes, such as job satisfaction, turnover intention, organization commitment, citizenship behavior, and job engagement (36–38).

The multidimensional fit (39) emphasizes that people do not interact with only one part of the environment (40) and there exist different fits satisfying different needs. Thus, several distinct measurements for P–E fit have been developed. Four types of the P–E fit are often measured: person-job (PJ) fit, person-group (PG) fit, person-supervisor (PS) fit, and person-organization (PO) fit (41). Specifically, PJ fit captures the congruence of an individual and his/her job position, and can be further divided into needs-supplies (NS) fit and demands-abilities (DA) fit; PG fit measures the compatibility of an individual and his/her team members, and can be further divided into supplementary [person-group supplementary (PGS)] fit and complementary (PGC) fit; PS fit refers to how compatible an individual was with his/her supervisor and PO fit measure the fitness of an individual and the organization he/her is working for. To fully understand fit of an individual in the work environment, studies have suggested that multiple dimensions of P–E fit should be integrated and examined (42, 43).

### Relationship Between P–E Fit and Academic Output

From the perspective of industrial and organizational psychology, both the requirement of academic publications for promotion and P–E fit falls into the category of incentive. The concept of incentive is developed to analyze and explain how to motivate people to work and achieve goals in organizations (44). Incentive can be categorized as intrinsic and extrinsic incentives (45). Intrinsic incentives can satisfy personal needs directly by creating an intrinsic reward for those who perform the tasks (46). It is consistent with the connotation of P–E fit theories. Value congruence influences outcomes through goals (motivation), when there is fit, the environment affords individuals with the opportunity to fulfill their needs (47). Extrinsic incentives are driven by the instrumental gain and loss, including pay and fringe benefits, gifts, promotion, advancement opportunities, etc. The practice of including academic output in promotion criteria, operated as a reinforcer, motivates clinicians to reprioritize their daily tasks. Meta-analysis has demonstrated that intrinsic incentives and extrinsic incentives have joint impact on performance and suggested both incentives be best considered simultaneously in term of performance study (48).

Despite a scattering of primary studies examining the differential effect of engaging in research and P–E fit independently on medical practice, few studies have considered these two strands of factors simultaneously under the framework of motivation in the real world. It is worth a try to pay more the attention to clinicians themselves in human resource management, thereby the promotion mechanisms may enable both wellbeing and fulfillment at work of health service employees and ultimately social efficiency in service delivery (49).

In this study, two strands of factors are taken into account: one is academic outputs driving by extrinsic incentive, and the other is the intrinsic incentive represented by P–E fit. As very few studies have examined the rationality of the clinical career progression in China (50), this study uses empirical data to assess academic outputs, P–E fit, or clinical performance of clinicians. By discussing the role of P–E fit and academic outputs in improving clinical performance, this study adds by providing a more comprehensive perspective on the evidence of incentives to the health workforce.

We explore two research questions regarding the promotion mechanism and the potential drivers to clinical JP:

1. Is there any difference of academic outputs, P–E fit, or clinical performance between clinicians who were promoted and those who were not?
2. Are academic outputs and P–E fit positively related to clinical performance?

## Materials and Methods

### Setting and Participants and Data Source

In Shanghai, clinicians who have met prerequisites determined by the certifying agency [Shanghai Health Personnel Exchange Service Center (SHPESC)] submit a promotion package. And then a qualification appraisal technical specialty committee will review the package, make decisions, and offer titles to the selected clinicians. The study sample included in this study was 426 wards clinicians who sought promotion to associate chief or chief doctors in ten pilot clinical specialties undergoing promotion reform from 67 public hospitals in Shanghai, China, 2020.

The personal information such as gender, age, education was obtained from SHPESC in charge of health resources management in Shanghai. The system contains not only basic demography information of physicians, but also information on duration in the current position, the number of academic papers, and the number of awarded RP during the current position. We obtained clinical information for clinicians from inpatient electronic medical record (EMR). All relevant clinical data, such as total surgeries performed, were obtained for the period of January 1, 2019 to December 31, 2019.

The P–E fit measures were collected through a questionnaire survey. Data were collected from October 1, 2019 to November 1, 2020. An encrypted access link of questionnaire was sent to clinicians together with administrative notification stating the appraisal process. Such practice guaranteed effective access of individual to the questionnaire. A detailed informed consent was given to them about the scope of the study. Each person was assigned a unique personal identification number for subsequent data matching. Privacy protection was also highlighted regarding the data transmission. The responses were directly uploaded to dedicated data server of the research group.

### Measures

To examine the relationship between clinical performance and P–E fit and academic outputs, we use multiple measures for each of these three dimensions.

#### Clinical Performance and Academic Outputs

We adopted objective JP measurements in this study and four dimensions of clinical performance were examined. They include: (1) quantity of services (QT) measured by the total number of surgeries and operations; (2) quality of services (QL) measured by the complexity of surgeries and procedures; (3) operating efficiency (EF) that includes both time efficiency (TE) measured by average LOS and cost efficiency (CE) measured by the hospitalization expense per patient; and (4) COs measured by the mortality rate. The measure of patient satisfaction mentioned in the part of “Assessment of clinical JP” was not considered in this study because satisfaction was a subjective feeling, which did not depend solely on the performance of clinicians, but was more susceptible to other factors, such as doctor–patient communication or treatment process.

The academic outputs in the promotion package included the number of awarded RP, the total number of published papers (TP), and the number of papers published as the first or corresponding author. The latter was considered publications as the main author (MP). To avoid the problem of collinearity in the subsequent regression model, the number of papers was calculated by subtracting MP from the total number of papers and it was identified as papers as a secondary author (SP). SP replaced TP in building the regression model.

The operational definition of clinical performance and academic outputs are shown in Additional Files. To balance the differences between specialty and disease, all indicators were normalized using the maximum difference normalization method by specialties, so that avoiding the situation that clinicians with the highest performance in one specialty would receive an unfair evaluation in other specialties. Then the indicators were transformed into their logarithmic form to adjust data distribution. According to the function of healthcare services, more scores on QT, QL mean better performance of the clinicians, and TE, CE, and CO in the opposite.

#### P–E Fit

Different types of P–E fit were measured using standard questionnaires, and all responses were anchored on a 5-point Likert scale, ranging from 1 = strongly disagree to 5 = strongly agree. A score for each type of fit was calculated by averaging the item score from each question.

**Person-job (PJ) fit** was measured using a 6-item scale developed by Cable and DeRue (51). There were three items on needs-supplies (NS) fit (e.g., “The attributes that I look for in a job are fulfilled very well by my present job”) and the other three on DA fit (e.g., “The match is very good between the demands of my job and my personal skills”). **Person-group (PG) fit** was measured by an adapted 9-item scale developed by De Cooman et al. (52). There were four items on supplementary (PGS) fit (e.g., “My skills and abilities match the skills and abilities this team looks for in team members”) and the other five on complementary (PGC) fit (e.g., “I feel that I am important to this team because I have such different skills and abilities than my team members”). **PS fit** was measured by a 6-item scale developed by Lankau et al. (53) (e.g., “I share similar values with my supervisor”). **PO fit** was measured by a 3-item scale developed by Cable and Judge (54) (“My personal values match my organization's values and culture”).

In terms of reliability and validity, several indicators of each P–E fit scales were examined. An item of PG fit (“My ability level is comparable to those of my team members”) was removed because of the non-significant factor loading (*t* = 0.054, *p* = 0.433) and poor model fit (χ^2^/*df* = 1.921, *p* = 0.011) in the validity analysis. After removing this item, the construct of PG fit was of good model fit (χ^2^/*df* = 1.522, *p* = 0.108). We did not remove another item of PG fit with factor loading lower than 0.5 (*t* = 0.355, *p* < 0.001) for two reasons. First, removing the items would leave only two items in PGS fit, which would result in <3 observable variables in this dimension. Besides, although the removal of this item improved the coefficient of Cronbach's α, composite reliability (CR), and average of variance extracted (AVE), it led to a poor model fit (χ^2^/*df* = 2.343, *p* = 0.016).

Of the four scales, the Cronbach's α ranged from 0.848 to 0.923 (>0.75), the CR ranged from 0.852 to 0.924 (>0.7), the AVE ranged from 0.431 to 0.801 (0.36–0.5 is acceptable while 0.5 is appropriate), showed that the measurements were of good reliability. Confirmatory factor analysis showed that the all the constructs of P–E fit were of good validity, with all the items statistically significant with factor loadings ranging from 0.355 to 0.922 (>0.5), Root Mean Square Error of Approximation (RMSEA) ranged from 0.000 to 0.046 (<0.08), comparative fit index (CFI) ranged from 0.993 to 1.000 (>0.9), goodness-of-fit index (GFI) ranged from 0.981 to 0.995 (> 0.9), adjusted goodness-of-fit index (AGFI) ranged from 0.944 to 0.976 (>0.9), χ^2^/*df* ranged from 0.771 to 1.522 (<2) with good model fit (*p* > 0.05).

#### Other Variables

We also obtained information on gender, education, specialty, management position (including head of the department, deputy department director, and vice president of the hospital), and duration in the current position of clinicians. Dummy variables were used for measuring education attainment and specialty. For education, degree of bachelor served as the reference category. There were 9 clinical specialties, and general surgery served as the reference category. Duration in the current position was measured in years and treated as a continuous variable. The professional title was not controlled because of the administrative directive restriction on main indicators. In addition, we found that age was highly correlated with duration in the current position (Pearson's correlation coefficient *r* = 0.674, *p* < 0.001). We only included the duration in the regression model since other studies concluded that job experience was more predictive of JP in high complexity jobs than age (49).

### Valid Sample and Exclusion

Those clinicians (1) who had short work histories (<2 years) in current hospital; (2) who had an incomplete medical record (e.g., had more than 20% missing values on the surgery level of the total number of surgeries or operations) were excluded from the analysis. Additionally, we also exclude the following individuals from the analysis: (1) those who had outliers exceeding five standard deviations on clinical performance and academic output indicators; (2) those who failed to submit P–E fit questionnaires, and (3) those whose answer time for P–E fit questionnaires was too short to be valid.

By the deadline of the data collection, we collected data for 259 clinicians, accounting for 60.80% of all clinicians (426) who applied for the promotion. We merged data on personal characteristics, academic outputs, P–E fit and clinical performance, and built a dataset for further statistical analysis. The final sample for this study included 244 clinicians. Considering the number of dummy variables eventually included in the regression model, we have 16 independent variables. Following the rule of thumb of having at least 10–20 events per variable (EPV) in the modeling process, we would need 160–320 samples. Thus, the sample size is sufficient (55, 56). The sample selection process and testing for non-response bias were shown in Additional Files.

### Statistical Analysis

A descriptive analysis was conducted for all variables used in this study, including demographic characteristics and indicators measuring academic outputs, PE fit, and clinical performance. Bivariate analysis to test the overall differences on all indicators between the two groups (those who got promoted and those who did not) was conducted through *t*-test and chi-square test. Several multi-regression models based on ordinary least squares were conducted to explore the association between academic outputs, P–E fit and five various clinical performances (QT, QL, TE, CE, and CO). We used clinical performance indicators as dependent variables and demographic characteristics, academic output indicators, and P–E fit indicators as independent variables. The independent variables were entered into the model as a group step by step, starting with the group of demographic characteristics, then academic outputs, and then P–E fit. For linear regression models, the collinearity of independent variables was tested and we used Variance Inflation Factors (VIF) of 5 as a cut-off (57) for collinearity.

To handle multiple sources of data, DBeaver 4.3.0 database management tool was used to access database, filter, and link records. SPSS software package program (SPSS for windows 7, version 21.0, SPSS Incorporation, Chicago, Illinois, USA) was used for statistical analysis. The threshold of statistical significance was set at 0.05 (two tailed).

## Results

The characteristics of clinicians who were promoted (190) compared to those who were not promoted (58) were presented in Table 1. The promotion rate was 77.9%. There was no statistical difference between the two groups in age, gender, specialty, and duration in the current position. The majority of clinicians were aged between 36 and 45 (74.2%), male (68.9%), surgeons (56.2%), and held their position for 6–15 years (84.4%). However, the degree and professional title distribution were different between the two groups. Those who were promoted were more likely to have a doctoral degree than those who were not. The differences were statistically significant (*p* < 0.001).

**Table 1:** The characteristics and main indicators of the promoted and unpromoted clinicians.

		**All (*N* = 244)** ***n* (%)/*M* (*IQR*)**	**Get promotion**		
			**No (*n* = 54)** ***n* (%)/*M* (*IQR*)**	**Yes (*n* = 190)** ***n* (%)/*M* (*IQR*)**	
Gender	Female	76 (31.15)	17 (31.48)	59 (31.05)	0.004
	Male	168 (68.85)	37 (68.52)	131 (68.95)	
Age (years)	32–35	17 (6.97)	3 (5.56)	14 (7.37)	1.008
	36–40	105 (43.03)	23 (42.59)	82 (43.16)	
	41–45	76 (31.15)	19 (35.19)	57 (30.00)	
	46–50	27 (11.07)	6 (11.11)	21 (11.05)	
	51–59	19 (7.79)	3 (5.56)	16 (8.42)	
Degree	Bechelor	85 (34.84)	32 (59.26)	53 (27.89)	26.732^***^
	Master	75 (30.74)	18 (33.33)	57 (30)	
	PhD	84 (34.43)	4 (7.41)	80 (42.11)	
Professional title	Attending doctor	189 (77.46)	50 (92.59)	139 (73.16)	9.096^**^
	Associate chief doctor	55 (22.54)	4 (7.41)	51 (26.84)	
Leadership position	No	204 (83.61)	48 (88.89)	156 (82.11)	1.412
	Yes	40 (16.39)	6 (11.11)	34 (17.89)	
Specialty	Surgery	137 (56.15)	30 (55.56)	107 (56.32)	0.377
	Internal	39 (15.98)	10 (18.52)	29 (15.26)	
	Gynecology & pediatric	68 (27.87)	14 (25.93)	54 (28.42)	
Duration in the current position (years)	3–5	14 (5.74)	1 (1.85)	13 (6.84)	3.958
	6–10	122 (50.00)	26 (48.15)	96 (50.53)	
	11–15	84 (34.43)	19 (35.19)	65 (34.21)	
	16–23	24 (9.84)	8 (14.81)	16 (8.42)	
Clinical performance	QT	253 (521.75)	214.5 (316)	278 (583)	−1.662
	QL	0.48 (0.20)	0.45 (0.18)	0.49 (0.20)	−1.493
	TE	9.27 (6.61)	9.22 (6.86)	9.33 (6.86)	0.167
	CE	30,788.23 (38,221.33)	27,096.57 (27,899.29)	32,856.62 (40,189.93)	−1.728
	CO	0.00 (0.00)	0.00 (0.00)	0.00 (0.00)	0.233
Academic output	TP	4.00 (3.00)	3.00 (3.00)	5.00 (3.00)	−5.075^***^
	MP	3.00 (5.00)	2.50 (4.00)	3.00 (5.00)	−1.543
	SP	0.00 (3.00)	0.00 (2.00)	0.00 (3.00)	−0.537
	RP	2.00 (3.00)	2.00 (3.00)	3.00 (3.00)	−3.429^**^
Person-environment fit	NS fit	4.00 (0.66)	4.00 (0.66)	4.00 (0.66)	−0.359
	DA fit	4.00 (0.67)	4.00 (0.67)	4.00 (0.67)	−0.268
	PGS fit	4.00 (0.92)	4.00 (0.66)	4.00 (1.00)	−0.364
	PGC fit	4.00 (0.60)	4.00 (0.65)	4.00 (0.60)	0.221
	PS fit	3.83 (0.50)	3.67 (0.84)	3.83 (0.50)	−0.24
	PO fit	4.00 (0.00)	4.00 (0.00)	4.00 (0.33)	−0.551

*The descriptive statistic for size was based on the absolute number and the statistical test for size was based on the transformed value after taking natural log and normalization*.

* *p < 0.05*,

** *p < 0.01*,

*** *p < 0.001*.

Regarding clinical performance, we found no differences between the two groups. However, for academic outputs, we found that those who were promoted had significant higher number of total papers (*t* = −5.075, *p* < 0.001) and higher number of RP (*t* = −3.429, *p* = 0.001) than those who were not. There was no difference in P–E fit between the two groups.

Table 2 presents the median, interquartile range (IQR), and Spearman's rank correlation coefficients of all the main variables in this study. The skewness and kurtosis of the main variables ranged from −1.151 to 5.289 and −1.277 to 34.346, respectively. There was correlation among some clinical performance indicators. For example, QT was positively correlated with QL (*r* = 0.401, *p* < 0.001) but negatively correlated with TE (*r* = −0.378, *p* < 0.001). CE was positively correlated with QL (*r* = 0.161, *p* = 0.012) and TE (*r* = 0.638, *p* < 0.001). CO was positively correlated with QL (*r* = 0.140, *p* = 0.029), TE (*r* = 0.239, *p* = < 0.001), and CE (*r* = 0.382, *p* = < 0.001). We also found that a few academic output indicators were associated with clinical performance indicators such as association of MP with CE (*r* = 0.160, *p* = 0.013) and CO (*r* = 0.143, *p* = 0.026) and association of RP with CE (*r* = 0.142, *p* = 0.026). Additionally, two P–E fit indicators were associated with clinical performance indicators. PGS fit was negative associated with CO (*r* = −0.142, *p* = 0.026) and PGC fit was positively associated with QL (*r* = 0.173, *p* = 0.007). Additionally, we found that there was some correlation among academic output indicators, but academic outputs were not correlated with any P–E fit variables. P–E variables were highly correlated among themselves.

**Table 2:** Spearman's rank correlation coefficient of clinical performance, academic outputs, and P–E fit.

		**Median**	**IQR**	**1**	**2**	**3**	**4**	**5**	**6**	**7**	**8**	**9**	**10**	**11**	**12**	**13**	**14**	**15**
Clinical performance	1. QT	5.533	2.002	–														
	2. QL	−0.734	0.426	0.401^**^	–													
	3. TE	2.227	0.695	−0.378^**^	−0.108	–												
	4. CE	10.335	1.219	−0.004	0.161^*^	0.638^**^	–											
	5. CO	0.000	0.004	0.117	0.140^*^	0.239^**^	0.382^**^	–										
Academic output	6. TP	1.609	0.560	0.121	0.196^**^	0.036	0.226^**^	0.103	–									
	7. MP	1.386	1.792	0.051	0.087	0.066	0.160^*^	0.143^*^	0.577^**^	–								
	8. SP	0.000	1.386	−0.006	0.036	−0.049	−0.038	−0.078	−0.006	−0.785^**^	–							
	9. RP	1.099	0.916	0.030	0.118	−0.008	0.142^*^	0.021	0.400^**^	0.382^**^	−0.205^**^	–						
Person–environment fit	10. NS fit	4.000	0.660	0.118	0.070	−0.086	−0.010	−0.079	0.030	−0.002	−0.013	0.070	–					
	11. DA fit	4.000	0.670	0.070	0.124	−0.104	0.017	−0.115	0.056	0.022	−0.013	0.091	0.679^**^	–				
	12. PGS fit	4.000	0.915	−0.017	0.092	0.031	0.029	−0.059	0.064	0.049	−0.045	0.096	0.563^**^	0.681^**^	–			
	13. PGC fit	4.000	0.600	0.075	0.173^**^	−0.027	0.083	0.006	−0.002	0.021	−0.063	0.036	0.382^**^	0.456^**^	0.468^**^	–		
	14. PS fit	3.830	0.500	0.020	0.034	−0.035	0.019	−0.095	−0.046	0.075	−0.130^*^	0.113	0.556^**^	0.526^**^	0.589^**^	0.402^**^	–	
	15. PO fit	4.000	0.000	−0.013	0.052	−0.109	−0.049	−0.142^*^	−0.041	0.046	−0.124	0.093	0.539^**^	0.582^**^	0.494^**^	0.366^**^	0.658^**^	–

*The descriptive statistic for size was based on the absolute number, and the statistical test for size was based on the transformed value after natural log and zero–score normalization. N = 244*,

* *p < 0.05*,

** *p < 0.01*.

Table 3 reports the results from the full model of the multiple regression analysis on drivers of clinical performance. The VIF of all the variables in the regression model were ranged from 1.014 to 4.290. Overall, the academic outputs explained no significant amount of variance of all the 5 models, and the P–E fit explained a significant amount of variance in CO (Model 5, Δ*R*^2^ = 0.081, Δ*F* = 3.568, *p* = 0.002).

**Table 3:** Regression results on the relationship between academic outputs, P–E fit, and clinical performance.

	**Model 1**	**Model 2**	**Model 3**	**Model 4**	**Model 5**
	**DV: QT**	**DV: QL**	**DV: TE**	**DV: CE**	**DV: CO**
**Step 1: Control variables**					
**Gender**					
Female	ref	ref	ref	ref	ref
Male	0.187^*^	0.065	0.132	0.334^***^	0.113
**Degree**					
MD	ref	ref	ref	ref	ref
Master	−0.036	0.036	0.062	0.132^*^	0.123
PhD	−0.013	0.087	0.024	0.254^***^	0.020
**Specialty**					
Surgery	ref	ref	ref	ref	ref
Internal	−0.195^**^	−0.196^**^	0.027	0.041	0.103
Gynecology & Pediatric	0.114	−0.059	−0.306	−0.314^***^	−0.048
**Leadership position**					
No	ref	ref	ref	ref	ref
Yes	−0.130^*^	−0.002	0.088	−0.096	0.002
Duration in the current position	0.029	0.062	0.033	0.003	−0.029
R^2^	0.096^**^	0.077^*^	0.170^***^	0.476^***^	0.044
**Step 2: Academic outputs**					
MP	0.235	0.214	0.014	0.178	0.181
SP	0.169	0.233	−0.068	0.098	0.062
RP	0.003	0.077	−0.052	−0.000	−0.106
Δ*R^2^*	0.013	0.020	0.006	0.009	0.022
**Step 3: P–E fit**					
NS fit	0.087	0.089	0.095	0.031	0.027
DA fit	0.169	0.068	−0.298^**^	−0.088	−0.291^**^
PGS fit	−0.220^*^	−0.086	0.141	0.003	−0.087
PGC fit	0.066	0.159^*^	0.009	0.092	0.136
PS fit	0.182	0.004	−0.091	−0.068	0.011
PO fit	−0.167	−0.051	0.058	0.050	0.023
Δ*R^2^*	0.068	0.034	0.036	0.009	0.081^**^
Total *R^2^* (Adj *R^2^*)	0.153 (0.094)	0.131 (0.070)	0.213 (0.157)	0.495 (0.459)	0.146 (0.086)
Total *F*	2.567^***^	2.145^**^	3.829^***^	13.899^***^	2.434^**^

*N = 244*,

* *p < 0.05*,

** *p < 0.01*,

*** *p < 0.001*.

In the final model, we found that QT was not associated with any academic output indicators but was negatively associated with PGS fit (β = −0.220, *p* = 0.025). QL was not associated with many academic output indicators but was positively associated with PGC fit (β = 0.159, *p* = 0.034). TE was not associated with any academic output indicators, but negatively associated with DA fit (β = −0.298, *p* = 0.002). A high level of DA was associated with a shorter LOS. CE was not associated with any academic output and P–E fit indicators. CO was not associated with any academic output indicators, but negatively associated DA fit (β = −0.291, *p* = 0.004). A high level of DA fit was associated with a low level of mortality rate.

Additionally, the results also suggested that gender, degree, and specialty of clinicians were associated with clinical performance. Male clinicians were associated with higher QT (Model 1: β = 0.187, *p* = 0.035) and CE (Model 4: β = 0.334, *p* < 0.001) than female. Clinicians with degree of master and PhD were associated with higher CE (Model 4: β_*MD*_ = 0.132, *p* = 0.027; β_*PhD*_ = 0.254, *p* < 0.001). Clinicians with leadership position were associated with lower QT (Model 1: β = −0.130, *p* = 0.044) than those who without. The results also showed specialty differences in various clinical performance indicators.

## Discussion

This study poses two questions. One is whether clinicians who are promoted are different from those who are not in academic outputs or clinical performance, and the second question is how academic outputs or P–E fit relates to clinical performance. For the former question, we find that people with more academic outputs have greater advantages in the promotion, and for the latter question, we find that P–E fit is more important in determining clinical performance than academic outputs. Overall, considered simultaneously through meta-analytic regression, intrinsic motivation predicted more unique variance in quality of performance, whereas incentives were a better predictor of the quantity of performance (48).

Clinicians who are promoted have more academic outputs than those who are not. It may relate to the promotion criteria of professional title that advocate for the academic performance of clinicians. Since most clinicians pursue an advanced title of the profession in the career ladder, they inevitably face a race on the pressure of publication (59). According to the goal-setting theory, the requirement of publication was clear and measurable because agents are motivated to exert more effort to achieve better performance (58), especially in the aspect of quantity performance (60). Consequently, it is reasonable to assume that such results, to some extent, were related to the specific goals of academic output in the promotion mechanism. This finding is in line with prior studies that acknowledged the definition of clear and measurable goals is positively associated with performance (61). Since clinical competency is often harder to show in a specific, quantitative way, and the promotion reviewers may be more sensitive to the measurable academic output, candidates are inclined to be involved in the more scientific activity to get a competitive advantage.

We do not find significant differences in various clinical performances among clinicians who are promoted or not. It is likely that the advantages of clinical performance are not so prominent in such a group of clinicians. Of the clinicians included in this study, they have won the competition within the hospital so that they are qualified for the appraisal process alongside an outstanding peer. In other words, they have already been at the top of the corresponding profession. Accordingly, they may have an advantage over their colleagues in the hospital, rather than their peers in the whole industry. A low agreement degree of the removed item of PG fit scale also supported the situation.

Another main finding is that we empirically support that P–E fit plays a more important part in improving clinical performance than academic output. Clinicians' P–E fit has a significant and robust impact on their clinical performance, notably on CO. Specifically, higher DA fit is related to improvement in TE and CO. Such finding confirms the dominant position of the competence (e.g., knowledge, skills, and attitudes) of clinician in the healthcare profession (62). This result is consistent with a study conducted in the USA, which shows that more experienced clinicians are associated with lower resource use and short-term mortality (63). The result is also consistent with an exploratory study that showed that poor PJ fit is associated with poor performance. Their study also find that clinicians with the higher PJ fit tended to participate in a variety of common clinical and non-clinical hospitalist activities (64). Such a tendency to participate in activities that need a wider set of their competencies may contribute to a better healthcare quality.

We also found that PG fit was directly associated with QT and QL. Meta-analytic results have confirmed the relationships between PG fit and subjective JP. PG fit has been related to both contextual performance and overall performance (33). Our findings are consistent with conclusion of the previous study that effective team processes have an effect on the successful provision of patient care (65). However, this study shows a counter-intuitive relationship between PGS fit and QT. Presumably, there are missing moderators, such as team traits or individual personality. For example, a meta-analysis conducts in Southeast Asia finds that heterogeneity on supplementary trait has different effect on the relationships between PG fit and JP. Teams with uniformly low in conscientiousness may agree on low levels of performance goals, effort, and planning which in turn are associated with low group performance.

## Limitations and Strengths

This study has several limitations. First, the results present correlations rather than causal relations among variables. In this study, the result of promotion is used to verify the performance disparity under the extrinsic motivation brought by the promotion mechanism. However, the promotion itself is expected to affect intrinsic motivation (66) because promotion often affects tasks people are assigned to, which releases a signal of trust and leads to empowerment (67). Hence, more research is therefore needed to track changes and validate the causal flow using longitudinal data. Second, we only include measures that could be obtained through EMR in the analysis, and this may result in the omission of some important but difficult to obtain performance indicators. For example, we were not able to obtain information on patient case mix in the assessment of clinical performance, which may lead to assessment biases. Lastly, there are some missing values when using different databases, and we have completed data for 57.28% of all applicants, and they are similar to the other 157 invalid samples on demography and main indicators.

Despite these limitations, this study has several strengths that merit attention. First, we use multiple data resources to measure variables of interest in the study, addressing concerns of heavily relying on a single resource, which may lead to common method bias. Second, the measurement of clinical performance is based on objective administrative records, which focus on observable, countable, discrete outcomes, avoiding the potential of suffering from overinflated assessment and self-serving bias. Third, by examining P–E fit and individual clinical performance, our practices add to the existing literature that P–E fit has a positive effect on the objective work-related outcome, not merely subjective JP.

## Conclusion

Healthcare human resource managers may be able to utilize the results of this study for selecting competent health personnel for better health outcomes for served populations.

First, the current promotion mechanism puts a higher weight on academic outputs, rather than a high quality of care. The appraisal criteria may need to be revised. This is consistent to the recent call in China to refocus on clinical competency for clinicians during the promotion process. Clear and measurable benchmarks for clinical performance and competency should be developed, and higher weight should be given to clinical measures. PMS and other incentive mechanisms should be refined by working with clinicians themselves, fostering their ownership of and engagement in the mechanisms (68), so as to lower their resistance to accepting externally imposed goals.

Second, this study does not support the assumption that academic outcomes contribute to the COs. Instead, P–E fit plays a more important role in COs. Thus, it is important to assess P–E fit during the hiring and creating an amiable environment for employees to improve quality health care in hospitals. For example, our analysis of the clinicals shows that longer periods without a realized or expected promotion are related to a decrease in P–E fit. Such finding is consistent with another Chinese study (69). Once attitudinal and behavioral variables are adequately stimulated in hospitals to bolster better P–E fit, the introduction of business-oriented techniques in the healthcare sector tends to play a great role in improving performance (70).

In general, this study shows that P–E fit plays a more important role in facilitating clinical performance than academic performance and highlights the importance of intrinsic motivation of clinicians in achieving clinical performance. For clinicians in public hospitals, they may have higher public services motivation than those employees in the private sector and are fundamentally motivated to serve the public interest (71), that is to provide higher quality services. Despite the orientation of more academic output, clinicians of the public hospital are primary true to their profession rather than the external conditions they are requested by.

## Data Availability Statement

The raw data supporting the conclusions of this article will be made available by the authors, without undue reservation.

## Ethics Statement

This study was approved by the Institutional Review Board of School of Public Health, Shanghai JiaoTong University School of Medicine, China on February 20, 2020, record number SJUPN-202008 using the National Statement on Ethical Conduct in Human Research. Data collection was conducted from October to November 2020.

## Author Contributions

MD and YX drafted the manuscript and performed the statistical analysis. MD contributed to organization and coordination. YX designed and administered the electronic version of the survey. CS provided methodological guidance. CS and WZ made critical revisions and additions to the manuscript. FW and GL were responsible for the research design, in charge of manuscript revision and project guidance. All authors contributed to the article and approved the submitted version.

## Funding

This research project was funded by a grant from the National Natural Science Foundation of China (Grant 72074147) and Philosophy and Social Sciences of the Ministry of Education of China (Grant 18JZD044).

## Conflict of Interest

The authors declare that the research was conducted in the absence of any commercial or financial relationships that could be construed as a potential conflict of interest.

## Publisher's Note

All claims expressed in this article are solely those of the authors and do not necessarily represent those of their affiliated organizations, or those of the publisher, the editors and the reviewers. Any product that may be evaluated in this article, or claim that may be made by its manufacturer, is not guaranteed or endorsed by the publisher.
