# Supplementary material for: Which Contributes to Clinical Performance: Academic Output or Person–Environment Fit?
Source: Front Public Health. 2022 Mar 4;10:801917. doi: 10.3389/fpubh.2022.801917 (PMC8931592; doi:10.3389/fpubh.2022.801917)
Supplement: Supplementary file 1 [file Data_Sheet_1.pdf]

Additional files

Table S1 Implications of main variables and corresponding operational definition

Variable	Implications	Operational Definition
Clinical performance		
Quantity of products or services (QT)	Total number of surgery and operation	$QT = \sum N_i$, for each clinician, the total number of surgeries or operations of specific role i he acted in is denoted as N_i, the role of operations including operator,1st assistant or 2nd assistant. Integer ranges from 0 through positive infinity.
Quality of product or services (QL)	Degree of difficulty calculated according to the ratio of surgeries for different levels and roles.	$QL = \frac{N_i \times W_i \times \frac{\sum N_{ij} \times w_j}{\sum N_{ij}}}{\sum N_i}$, each surgery or operation is assigned a level of difficulty, ranged 1 through 4, and level 4 is the most difficult. The total number of surgeries or operations for level j is denoted as N_j, and the corresponding number of surgeries of each level for specific role is N_{ij}. W_i and w_j represent the weight assigned to each surgical role and surgical grade, respectively. Ranges from 0 through 1.
Time efficiency (TE)	Average LOS per patient (days)	$TE = \frac{\sum LOS_m}{N_m}$, for each clinician, the total number of patients that he/she handles is denoted as N_m, and the LOS for patient m is LOS_m. Days range from 0 through positive infinity.
Cost efficiency (CE)	Hospitalization expense per patient (CYN)	$CE = \frac{\sum C_m}{N_m}$, for each clinician, the total number of patients that he/she handles is denoted as N_m, and the hospitalization expense for patient m is C_m. CYN ranges from 0 through positive infinity.
Clinical outcome (CO)	In-patient mortality rate	$OE = \frac{N_{dead}}{N_m}$, for each clinician, the death toll of his/her patient is denoted as N_{dead}. Ranges from 0 through 1.
Academic output		

Number of total papers (TP)	The total number of papers during current term	The time period varies from person to person according to educational level. Integer ranged from 0 through positive infinity.
Main author paper (MP)	The total number of papers published as the first or corresponding author during current term	
Secondary author paper (SP)	The total number of papers published as co-author during current term	
Research project (RP)	The total number of research projects during current term	

For testing the potential bias of non-response, we compared the differences in demography and main indicators between the valid and invalid samples, see in Table S2. Only QT was different between the two groups. The valid sample were more likely to perform more surgeries than those who were invalid (t=-2.149, p=0.032). We further tested the difference between the four groups which were divided by whether included in the study or get promoted, see in Table S3 and Table S4. The Kruskal-Wallis *H* showed that the distribution of QT was not identical among the four groups (F=2.568, p=0.054). And the results of Post Hoc Tests using Bonferroni methods showed that the distribution of QT was different between any two groups (p value ranged from 0.191 to 1.000). Despite the differences between the valid and invalid samples, the distribution of QT was same between those who were promoted or not, such response bias would not affect the subsequence analysis.

Table S2 Testing for non-response bias on the characteristics and main indicators of the valid and invalid samples of questionnaire

		All (N=401)<br>n (%)/M (IQR)	Valid sample		χ^2/t<br>value
			No (n=157)<br>n (%)/M (IQR)	Yes (n=244)<br>n (%)/M (IQR)	
Gender	Female	113(28.18)	37(23.57)	76(31.15)	2.713
	Male	288(71.82)	120(76.43)	168(68.85)	
Age (years)	32-35	22(5.49)	5(3.18)	17(6.97)	2.846
	36-40	177(44.14)	72(45.86)	105(43.03)	
	41-45	126(31.42)	50(31.85)	76(31.15)	
	46-50	46(11.47)	19(12.1)	27(11.07)	
	51-59	30(7.48)	11(7.01)	19(7.79)	
Degree	Bachelor	124(30.92)	39(24.84)	85(34.84)	5.364
	Master	124(30.92)	49(31.21)	75(30.74)	
	Doctor	153(38.15)	69(43.95)	84(34.43)	
Profession al title	Attending doctor	298(74.31)	109(69.43)	189(77.46)	3.229

	Associate chief doctor	103(25.69)	48(30.57)	55(22.54)	
Specialty	Surgery	61(59.85)	22(65.61)	39(56.15)	3.819
	Internal	240(15.21)	103(14.01)	137(15.98)	
	Gynecology & pediatric	100(24.94)	32(20.38)	68(27.87)	
Duration in the current position (years)	3-5	27(6.73)	13(8.28)	14(5.74)	2.315
	6-10	200(49.88)	78(49.68)	122(50.00)	
	11-15	140(34.91)	56(35.67)	84(34.43)	
	16-23	34(8.48)	10(6.37)	24(9.84)	
Get Promoted	No	157(23.54)	100(24.63)	57(21.84)	0.688
	Yes	510(76.46)	306(75.37)	204(78.16)	
Clinical performance	QT	238(463.50)	198(430.5)	253(521.75)	-2.149*
	QL	0.48(0.21)	0.5(0.23)	0.48(0.2)	1.348
	TE	9.12(7.02)	8.81(7.54)	9.27(6.61)	0.066
	CE	32158.62(37705.25)	33452.64(36644)	30788.23(38221.33)	1.114
	CO	0.00(0.00)	0.00 (0.00)	0.00 (0.00)	0.887
Academic output	TP	5.00 (3.00)	5.00 (4.00)	4.00 (3.00)	1.444
	MP	3.00 (5.00)	3.00 (5.00)	3.00 (5.00)	0.223
	SP	0.00 (3.00)	0.00 (3.00)	0.00 (3.00)	0.968
	RP	2.00 (3.00)	3.00 (3.00)	2.00 (3.00)	0.695

Note. The descriptive statistic for size were based on the absolute number, and the statistical test for size were based on the transformed value after taking natural log.

Clinical performance: QT=quantity of products or services provided; QL=quality of product or services provided; TE=time efficiency; CE=cost efficiency; CO=clinical outcomes. Academic output: TP= total papers; MP=main author papers; SP=secondary author papers; RP=research projects.

*p<0.05, **p<0.01, ***p<0.001

Table S3 Kruskal-Wallis *H* test between four groups which were divided by whether included in the study or get promoted

	Sum of Squares	df	Mean Square	*F*	*p* value
Between Groups	24.128	3	8.043	2.568	0.054
Within Groups	1243.138	397	3.131		
Total	1267.266	400			

Table S4 *p* value of Post Hoc Tests using Bonferroni methods to test the difference between the four groups

	Median	IQR	0	1	2	3
1. Invalid and not get promoted	150.00	561.00	-			
2. Valid but not get promoted	214.50	316.00	1.000	-		
3. Invalid but get promoted	203.00	413.00	1.000	1.000	-	
4. Valid and get promoted	278.00	583.00	0.278	0.664	0.191	-

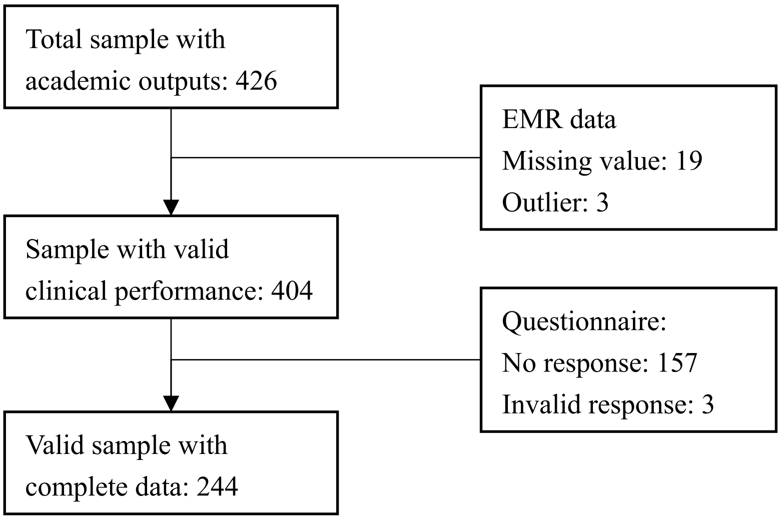

Figure S1 Sample selection process
